# Assessment of oral health status and treatment needs among people of Foklyan area, Dharan, Nepal

**DOI:** 10.1186/s12903-020-01312-2

**Published:** 2020-11-11

**Authors:** A. Singh, A. Shrestha, T. K. Bhagat, D. D. Baral

**Affiliations:** 1 Narayani Hospital, Birgunj, Parsa, Nepal; 2 Department of Public Health Dentistry, B. P. Koirala Institute of Health Sciences, Dharan, Nepal; 3 School of Public Health and Community Medicine, B. P. Koirala Institute of Health Sciences, Dharan, Nepal

**Keywords:** Sub-urban population, Nepal, Oral diseases, Oral health assessment, WHO oral health survey

## Abstract

**Background:**

Oral diseases are a major public health problem globally due to high prevalence and significant social impact. Foklyan is a peri urban area with people belonging to indigenous population of low socioeconomic status. This study was conducted to assess the oral health status and treatment needs among the people of Foklyan area, Dharan.

**Methods:**

Cross-sectional house to house survey was conducted on 310 randomly selected participants. The participants were stratified into five age groups as per WHO Basic Oral Health Survey Methods 1997 and further categorized by gender. WHO Oral Health Assessment form 1997, WHO oral health assessment questionnaire for adult/children 2013 and questionnaire for oral hygiene practice and cost as a treatment barrier were used. The examinations were done as per WHO standard guidelines.

**Results:**

Most of the participants were from low socioeconomic background (71.3%). About 40% of the participants deferred dental visit due to financial burden. Although 99% of the participants brushed their teeth, there was high caries experience (DMFT: 3.18 ± 5.85; dft: 2.40 ± 2.65). Mean sextant score for bleeding was 5.58 in 35–44 years age group and 5.61 in 65–74 years age group. Tobacco consumption was seen in 70.9% of the adults. Prevalence of alcohol consumption was 58.8% among adult age groups.

**Conclusion:**

The prevalence of dental caries, periodontal diseases, and prosthetic needs were more compared to national data. There is a need for oral health promotion in this area.

## Background

Oral health is a standard of the oral and related tissues which enables an individual to eat, speak, and socialize without active disease, discomfort, or embarrassment and which contributes to general wellbeing [1]. Oral health is a critical but overlooked component of overall health and wellbeing among children and adults. Oral diseases have a significant impact on the health and wellbeing of an individual through pain, morbidity, mortality, and the lost capacity to undertake school, social and economic activities.

In Nepal, ever since the commencement of National Oral Health policy in year 2004 A.D. there has been significant amount of dental problems identified but still at present the prevention and treatment of oral diseases is virtually unavailable to the rural and underprivileged population due to various educational, cultural and socioeconomic burdens [2]. The burden of oral disease due to associated pain and discomfort may result in loss of teeth, difficulty in eating, poor diet and consequently affect one’s appearance, self-esteem, and quality of life. There is a greatly increased risk of the development of life-threatening sequelae to dental infections due to poor nutrition, chronic diseases and the lack of availability of oral health care. Socioeconomic status, occupation, and education are playing a major role in the maintenance of good oral health. The maximum burden of all diseases rests with the disadvantaged and socially marginalized [3].

Dental caries is considered a major public health problem globally due to its high prevalence and significant social impact. World Health Organization reports 60–90% of schoolchildren worldwide have experienced caries, with the disease being most prevalent in Asian and Latin American countries [4]. In Nepal, the caries prevalence is found to be 64% in urban area and 78% in rural population whereas approximately 31% of age group 35–44 years have a deep periodontal pocket [2]. The WHO oral health assessment form 1997 is universally accepted for collection of all the information needed for planning oral care services and thorough monitoring and re-planning of existing care services.

Little is known about oral health attitudes and behaviors of people from developing countries as compared with developed countries [5]. There are few pieces of literatures conducted on the assessment of oral health status, oral health behavior among such underprivileged population in Nepal. Dental caries, gingivitis and periodontitis are very common oral health problems of Nepalese population, and the severity is more in case of underprivileged indigenous population groups [2]. Nepal is a poor underdeveloped country in terms of availability and accessibility of resources. Foklyan is a peri urban area of Dharan Sub-metropolitan city with most of the people belonging to the indigenous population of low socioeconomic status [6]. Studies have shown that the oral health of such population is poor and their attitude and practice towards oral health hygiene is often neglected [4, 7]. Hence, this study was conducted to assess the oral health status and treatment needs among the people of Foklyan area, Dharan.

## Methods

### Study design and participants

**Fig. 1:**
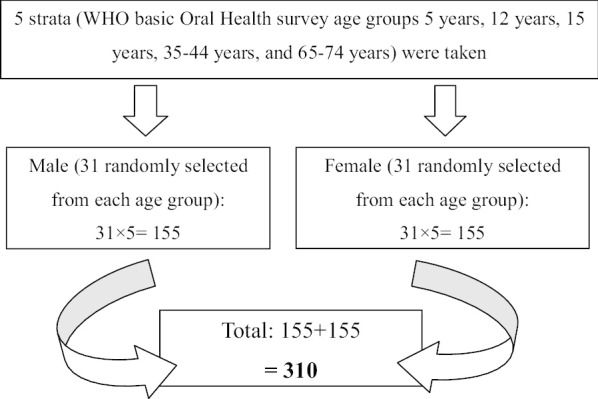
Flow diagram of the participants

It was a community-based cross-sectional study. The study was conducted from January 2017 to December 2017 among people of Foklyan area, Dharan, Nepal. Ethical approval was obtained from the Institutional Review Committee, BPKIHS, Dharan (Ref. No.: 300/074/074-IRC). Three hundred ten people were randomly selected. A stratified random sampling technique was used. The participants were stratified into five age groups as per WHO Basic Oral Health Survey Methods 1997 and further categorized by gender. Social mapping was done to select the houses fitting as per the sampling design. Thirty-one male and thirty-one females, for each age group, were randomly selected via lottery method from the 410 households in Foklyan. (Fig. 1).

This study considered (95% CI) to estimate the sample size. For this purpose, 60.3% prevalence of dental caries in eastern Nepal (p) was considered, and the calculated sample size was 256 [8]. To increase the sensitivity and validity, and considering 15% non-response and also to fit the study design sample size was increased to 310. The study included people above 5 years of age regardless of their medical condition (except psychiatric illness), and those not willing to take part in the study were excluded.

### Survey instruments

a WHO Oral Health Assessment form 1997: It is universally accepted and used recording methodology for oral health surveys. The form includes many sections, like, survey identification information, general information, extra-oral examination, TMJ assessment, oral mucosa, enamel opacities/hypoplasia, dental fluorosis, community periodontal index, loss of attachment, dentition ststus and treatment needs, prosthetic status/need, dentofacial anomalies, need for immediate care and referral [9].
b Questionnaire for oral hygiene practice and cost as a treatment barrier: These questions were formulated by the authors referring to various published literatures.
c WHO oral health questionnaire for children/ adult 2013: These questionnaires provide reliable information about oral health status and risk to oral health. There are separate sets of questionnaires for adult and children. Both questionnaires have been pilot-tested in range of countries across the world [10].
d Socioeconomic status: The Kuppuswamy’s Socioeconomic Status scale includes education, occupation of head of the family and income per month from all sources. It is categorized as upper class, upper middle class, lower middle class, upper lower class, and lower class. Kuppuswamy’s Socioeconomic Status scale was calculated and classified as per the modifications done in the year 2009 using the current consumer price index for the year 2017 [11, 12]. Education and Occupation does not change as per time but the income varies with time. So, during modification of Kupuswamy Scale, the income was re-categorized based on current consumer price index of year 2017. To re-categorize the income, the current consumer price index was obtained online from Nepal Rastra Bank website [12] and the conversion factor was calculated (Conversion factor = Consumer Price Index 2017 divided by Consumer Price Index 1976). The computed conversion factor was 26.7 (144.8/4.3). For simplicity, the classes were re-categorized as upper class, middle class and lower class [11].

### Survey procedure and outcome variables

Interview of the participants were conducted during the home visits. In case of children, either of the parents gave the interview. Time taken for each interview was 15–20 min. All the participants were examined at their houses by a single trained and calibrated examiner, under natural light using plane mouth mirror and WHO periodontal probe. Time taken for each examination was 5–10 min. The examinations were done as per WHO standard guidelines. Duplicate examinations were performed among 25 participants during the study to test the intra-examiner reliability. A pilot study was also conducted prior to the main study among 25 participants of similar community for training and calibration of the examiner along with the feasibility assessment of the study. Intra-examiner reproducibility was evaluated using Cohen Kappa statistics which showed excellent agreement (K = 0.912).

The primary outcomes measured were prevalence of dental caries, periodontal status and oral lesions along with oral hygiene behavior and cost as a barrier of participants. Data on socioeconomic status, enamel opacities, prosthetic needs, temporomandibular joint disorders, dentofacial anomalies and dental aesthetic index were also obtained. Dietary habits, tobacco consumption, alcohol consumption, pain and discomfort were also accessed.

### Data management and statistical measures

Data obtained were entered in Microsoft Excel Sheet 2007 and statistical analysis was done in SPSS version 11.5 (SPSS, Inc., Chicago, IL, USA) software. Descriptive statistics including the mean and standard deviations were computed for DMFT/dmft. Frequency distribution among the study population was calculated. Frequency of oral lesions, prosthetic status and prosthetic needs, community periodontal index and loss of attachment, dentition status and treatment needs, dental aesthetic index were assessed. Mann–Whitney test was used to compare the mean DMFT/dft. A *p* value < 0.05 was considered statistically significant.

## Results

The majority of the participants belonged to *Dalit* and *Janajati* category and about 12.3% belonged to *Brahmin–Chhetri* category as classified by Nepal Demographic and Health Survey [13]. Most of the people belonged to the lower class (71.3%) followed by the middle class (1.6%) and about 27.1% did not respond to socioeconomic status. Majority (82.3%) of the participants’ self-perception regarding oral health status was good to average.

It was seen that 99% of the participants brushed their teeth. Most of the participants brushed once a day or 2–6 times a week and it was seen that majority of the participants brushed for 3–5 min. Most of them were found to be brushing with their tooth brush (85.8%), followed by chewing stick (23.8%). Very few were seen using thread and wooden toothpicks for cleaning (0.9%). One participant was found using salt for cleaning teeth. Out of 99% who brushed their teeth, 89.1% used toothpaste. Table 1 (oral hygiene practices).

**Table 1:** Oral hygiene practices

	5 years	12 years	15 years	35–44 years	65–74 years	Total (%)
Brushing status						
Male						
Yes	25	30	31	29	23	138 (98.9)
Female						
Yes	25	28	31	31	26	141 (99.1)
Duration of tooth brushing						
1–2 min	17	11	8	5	11	52 (18.6)
2–3 min	8	20	20	11	10	69 (24.7)
More than 3 min	25	27	34	44	28	158 (56.7)
Frequency of tooth brushing						
Never	14	10	5	2	12	43 (13.8)
Once a day	1	1	3	31	16	52 (16.7)
Twice a day	0	0	0	5	3	8 (2.5)
1–3 times/week	47	50	54	22	27	200 (64.6)
1–3 times/month	0	1	0	2	4	7 (2.4)
Rinse of mouth after each meal						
Always	12	35	37	20	27	131 (42.4)
Sometimes	44	25	25	42	35	171 (54.9)
Never	6	2	0	0	0	8 (2.7)
Material used for tooth brushing						
Toothbrush	48	52	58	60	48	266 (85.8)
Chewing stick	1	3	9	26	35	74 (23.8)
Toothpick	0	0	0	1	1	2 (0.6)
Others	0	0	0	1	0	1 (0.3)

More than 60.0% of the males and females were having toothache/discomfort in the past 12 months but only 27.7% of males and 40.6% of females had a dental visit in their lifetime. Among the ones who had a dental visit, pain in the teeth was the main reason for the visit. Nearly 40% deferred dental visit due to financial problems. Out of those who visited the dentist, 60.6% of the participants were not able to afford the treatment and had to defer. Restorative treatments were deferred most. Table 2 (Dental problems and financial burden).

**Table 2:** Dental problems and financial burden

	5 years	12 years	15 years	35–44 years	65–74 years	Total (%)
Toothache/discomfort of participants in past 12 months						
Male						
Yes	20	17	16	15	26	94 (60.7)
No	8	11	10	7	4	40 (25.8)
Don’t know	3	3	5	9	1	21 (13.5)
Female						
Yes	13	17	19	24	25	98 (63.4)
No	13	9	10	6	2	40 (25.7)
Don’t know	5	5	2	1	4	17 (10.9)
Dental visit of participants in their lifetime						
Male						
Yes	1	8	13	12	9	43 (22.7)
Female						
Yes	7	10	18	14	14	63 (40.6)
Reason for dental visit						
Pain	4	14	23	14	16	71 (66.9)
Treatment	0	2	1	10	4	17 (16.0)
Routine checkup	4	2	7	2	3	18 (16.1)
Deferred dental visit due to financial burden						
Male	11	10	13	14	9	57 (46.7)
Female	8	13	13	13	18	65 (53.3)
Deferred dental treatment due to financial burden						
Male	1	6	9	10	7	33 (43.4)
Female	3	6	12	10	10	41 (56.6)
Types of treatment deferred						
Restorative	4	12	16	15	11	58 (80.6)
Prosthesis	0	0	0	0	7	7 (9.6)
Extraction	0	0	0	0	1	1 (1.4)
Scaling	0	0	1	5	0	6 (8.3)

The tobacco exposure was high in the adult age groups. More than 70% belonging to the age group 35–44 years and 65–74 years were consuming tobacco in either form (smokeless or smoke form). The participants in 15 years age group were also consuming tobacco (43.5%). The alcohol consumption in adults belonging to the age group 65–74 years was 54.8%.

The extra-oral status of all the participants was found normal. Overall, only six participants were having temporomandibular joint (TMJ) symptoms and on examination it was seen that 17 (0.5%) had clicking. On examination for oral mucosal lesions, five of the participants had abscess, six had tobacco pouch keratosis, one had leukoplakia, and six had ulceration. The lesions were seen in lips, sulci, buccal mucosa, floor of the mouth, and alveolar ridges.

Enamel hypoplasia with diffuse opacity was found in only one participant of 11 years of age, whereas very mild dental fluorosis was seen in 4 (1.3%) participants. When examined for prosthetic status, one had a bridge on the upper arch, and three participants had full removable denture on both upper and lower arches. Prosthetic needs were present in 23.6% for the upper arch and 21.7% for the lower arch. Pain and infection were seen in three participants and immediate referral was needed for one participant for abscess management. While assessing for Dental Aesthetic Index, elective treatment was indicated in 9.6% (category 2), highly desirable in 1.3% (category 3) and mandatory in 5.8% of the participants.

The periodontal status was assessed using the Community Periodontal Index (CPI) in adult age groups. Sextant with pocket depth 4–5 mm was seen in 11.1% participants of age group 35–44 years and 24.5% of age group 65–74 years. Mean sextant was more for calculus and was most prevalent, with 82.3% in 35–44 years age group and 74.5% in 65–74 years age group. Loss of attachment of 0–3 mm was more commonly observed in 35–44 years age groups (66.1%) and 4–5 mm in 65–74 years age group (43.6%). Loss of attachment of 6–8 mm was seen in 30.9% of the 65–74 years age group where as it was only in 6.4% in 35–44 years age group. Loss of attachment of 9–11 mm was also seen in six participants in both age groups. (Table 3).

**Table 3:** Periodontal status (CPI)

Distribution of study population based on the specific CPI scores (as per highest scores obtained)				
Age group	Healthy (H)	Bleeding (B)	Calculus (C)	Pocket 4–5 mm (P_1_)
35–44 years (n = 62)	2 (3.3%)	2 (3.3%)	51 (82.3%)	7 (11.1%)
65–74 years (n = 55)	0	0	41 (74.5%)	14 (24.5%)

Distribution of mean sextant CPI scores in each age group					
Age group	Healthy (0)	Bleeding (1)	Calculus (1 + 2 + 3 + 4)	Pocket 4–5 mm (2 + 3 + 4)	Excluded sextant (X)
35–44 years (n = 62)	0.4 (25)	5.58 (346)	5.40 (335)	0.22 (14)	0.01 (1)
65–74 years (n = 55)	0.05 (3)	5.61 (309)	5.5 (307)	0.34 (19)	0.23 (14)

Distribution of study population based on loss of attachments (as per highest scores obtained)				
Age group	LOA 0–3 mm (0)	LOA 4–5 mm (1)	LOA 6–8 mm (2)	LOA 9-11 mm (3)
35–44 years (n = 62)	41 (66.1%)	14 (22.5%)	4 (6.4%)	3 (4.8%)
65–74 years (n = 55)	11 (20.0%)	24 (43.6%)	17 (30.9%)	3 (5.4%)

Distribution of mean sextant loss of attachments in each age group					
Age group	LOA 0–3 mm (0)	LOA 4–5 mm (1)	LOA 6–8 mm (2)	LOA 9–11 mm (3)	Excluded sextant (X)
35–44 years (n = 62)	5.25 (326)	0.45 (28)	0.23 (14)	0.07(4)	–
65–74 years (n = 55)	3.49 (192)	1.05 (58)	0.98 (54)	0.22 (12)	0.26 (14)

Tooth decay was seen in 57.5% of people with permanent dentition, 28.4% of people with mixed dentition, and 65.1% of people with primary dentition. Missing due to caries was also more prevalent (34.4%) in people with permanent dentition followed by missing due to any other reason. Most of the participants had unexposed roots (71.5%) in permanent dentition.

One surface filling was required for 35.5% of the participants with permanent dentition, followed by 44.1% with primary dentition and 26.6% with mixed dentition. Extraction was indicated in 30.6% of the participants with permanent dentition, followed by 11.6% with primary dentition. Pulp care was needed mostly for participants with primary dentition (27.9%) followed by 12.3% of permanent dentition. (Table 4).

**Table 4:** Dentition status and treatment needs

Dentition status						Treatment needs			
Code and criteria^a^	Crown status			Root status		Scores^b^	Primary dentition	Mixed dentition	Permanent dentition
	Primary dentition	Mixed dentition	Permanent dentition	Mixed dentition	Permanent dentition				
0	14 (32.5%)	2 (2.5%)	4 (2.2%)	22 (27.2%)	185 (99.0%)	0	14 (32.5%)	7 (8.6%)	42 (22.6%)
1	28 (65.1%)	23 (28.4%)	107 (57.5%)	49 (60.5%)	2 (1.1%)	1	19 (44.1%)	20 (26.6%)	66 (35.5%)
2	7 (16.2%)	–	–	7 (8.6%)	–	2	17 (39.3%)	–	17 (9.1%)
3	–	–	6 (3.2%)	–	–	3	–	–	3 (1.6%)
4	–	–	64 (34.4%)	2 (2.5%)	–	4	–	–	–
5	2 (4.6%)	1 (1.2%)	46 (24.7%)	7 (8.7%)	–	5	12 (27.9%)	4 (4.9%)	21 (12.3%)
6	–	–	–	4 (4.9%)	–	6	5 (11.6%)	1 (1.2%)	57 (30.6%)
7	–	–	1 (0.5%)	–	–	7	–	–	62 (33.3%)
8	–	74 (91.4%)	133 (71.5%)	–	–	8	–	3 (3.7%)	105 (56.5%)
9	41 (95.3%)	42 (51.9%)	143 (76.9%)	61 (73.3%)	1 (0.5%)	9	–	2 (2.4%)	4 (2.1%)

^a^Code and criteria: 0: sound; 1: decay; 2: filled with decay; 3: filled with no decay; 4: missing due to caries; 5: missing due to other reason; 6: fissure sealant; 7: Bridge abutment, special crown; 8: unerupted tooth/crown, unexposed root; 9: not recorded

^b^Scores: 0: no treatment; 1: one surface filling; 2: two or more surface filling; 3: crown of any reasons; 4: veener and laminate; 5: pulp care and restoration; 6: extraction; 7: prosthesis; 8: oral prophylaxis; 9: not recorded

Mean DMFT among males was 3.14 ± 9.59, and that among females was 3.23 ± 5.76. The difference was not statistically significant (*p* = 0.720). Also, mean dft among males and females was not statistically significant (*p* = 0.400), the value being 2.64 ± 2.86 and 2.17 ± 2.416, respectively.

## Discussion

This study assessed the oral health status and treatment needs of Foklyan area, Dharan, Nepal. The study showed that more than 90% of the study participants cleaned their teeth. Use of tooth brush was found in 85.8% of the study population which is similar to the study conducted by Thapa et al. [14].

The impact of oral diseases, especially in an under privileged indigenous population, affects their lives influencing eating, sleeping, and working along with social responsibilities. It was seen that majority of the study population perceived their oral health as good to average despite a high prevalence of plaque, calculus, and dental caries. This might be due to the poor knowledge regarding the understanding of oral conditions and diseases. The findings were similar to the findings by Handa et al. [15] and Ghambhir et al. [16].

Toothache/discomfort was reported by more than 60% of the study population, but only 34.1% were found visiting the dentist for the problem. This can be well understood by the fact that most of the study population belonged to a lower socioeconomic class. Among the ones who visited, most were seen to visit due to pain in their teeth. Pain is the most common presentation of the dental patient visiting the dentist as reported in various studies [17, 18]. Nepal is a poor country with most of the population belonging to the low socioeconomic status mostly indigenous population [13]. The dental visit was deferred by 39.3% of the study population due to financial burden.

Use of the tobacco products was seen in 43.5% of the 15 years age group (smokeless or smoke form) where as in adult age groups more than 70.6% were found consuming tobacco products. Almost 60.0% of the adults in the age group 35–44 years and 65–74 years were seen consuming alcohol, where most were consuming at least 2 drinks each time of consumption. Most part of Nepal has cold climatic condition. Alcohol has been socially and culturally accepted among many ethnic groups such as *Janajatis* in Nepal. Population groups who do not belong to this ethnicity also have been found to be increasingly consuming alcohol [14].

The lesions observed on examination were very few cases of abscess, tobacco pouch keratosis, ulceration, and leukoplakia seen on the sulci, buccal mucosa, and floor of the mouth, and alveolar ridges. The most common lesion was tobacco pouch keratosis reflecting the high use of tobacco (70.0%) among the study population. These findings were in accordance with the study done by Bansal et al. [19]. Oral mucosal lesions could be due to infection, local trauma, or irritation (keratosis, fibroma etc.), systemic diseases or related to lifestyle factors such as use of tobacco, areca nut or alcohol.

Teeth in good condition are fundamental for a healthy oral environment and the body as a whole. Missing teeth lead to compromised mastication, speech, and facial appearance that affect the overall physical and physiological status of an individual. It was seen that only four participants had prosthesis whereas about 25% needed prosthesis in the upper arch and 30% needed prosthesis in the lower arch. Less use of prosthesis among the edentulous might be due to a lack of knowledge regarding the importance of the prosthesis and the poor socioeconomic status of the individuals. Similar kinds of findings were reported by the various studies [20–22].

About 3% of the participants in the 35–44 years age group had healthy sextant. Sextant with pocket depth 4–5 mm was seen in 11.1% participants of age group 35–44 years and 24.5% of age group 65–74 years. Despite the high prevalence of brushing, sextant with calculus was most prevalent, with 82.3% in 35–44 years age group and 74.5% in 65–74 years age group. This might be due to irregular brushing practice and improper technique of brushing [23]. About 40% of the study populations were seen to be brushing 2–6 times per week. Loss of attachment of 4–5 mm was observed in 35–44 years age groups (22.5%) and 4–5 mm in 65–74 years age group (43.6%) suggestive of periodontitis. The results were in accordance with the finding of the National Oral Health Pathfinder Survey where it was seen that 31% of the population belonging to the age group 35–44 years were having periodontitis [2]. Similar findings were also seen in the studies conducted by Goel et al. [24] and Varenne et al. [25]. The high tobacco consumption (70.6%) in adult study participants might be the contributing to high prevalence of periodontal diseases among that study participants [26].

The study showed that 65.1% had decay in their primary dentition, 28.4% of decay was seen in mixed dentition, and 57.5% of tooth decay in permanent dentition, while recording for crown status. The prevalence of dental caries (dft) in primary dentition was 65.1%, which was similar to the finding by Bhagat et al. [8], and the prevalence of dental caries in participants with permanent dentition (DMFT) was 75.2%. The mean dft was 2.40 and the mean DMFT was 3.18. The prevalence of dental caries was different than the National Oral Health Pathfinder Survey 2004 where dental caries prevalence was reported as 58.0%. The National Oral Health Pathfinder Survey was conducted more than a decade ago with an inclusive population of different ethnic communities whereas; these study participants mostly belonged to the underprivileged indigenous community. The neglected behavior of such a population towards oral health, irregular brushing and improper brushing technique leads to high caries and periodontal disease [27].

This study had some limitations. Since questions were asked about socioeconomic status, oral hygiene practice, discomfort/problems, probable chances of recall bias may have occurred because of the long time frame. Some of the respondents (27.1%) were reluctant to give their income status so no responses were obtained even after multiple attempts. The study was conducted in Foklyan area and the finding cannot be generalized to the population of other areas. A larger survey is needed to explore further but this study can be considered as a reference for planning oral health programs for similar population.

## Conclusion

This study concluded that most of the people of Foklyan area brush their teeth but not on a regular daily basis. There is a need for oral health promotion in this area that will enable them to better understand the importance of oral health. High prevalence of tobacco consumption among adult population and even half of the adolescent population already consuming tobacco products is an alarming issue. Appropriate tobacco control measure is must to control rampant tobacco use. Most of the study population belonged to the poor socioeconomic status; hence the financial burden of the oral diseases has added a detrimental effect on the individuals’ health. This is the first study of its kind done to explore the oral health status targeting the under privileged indigenous community. Hence, this study gives an idea to the policy makers to focus on the preventive aspects to minimize the oral health burden of the marginalized.

## Data Availability

The data supporting the findings of this article are available from the corresponding author.

## Abbreviations

BPKIHS: B. P. Koirala Institute of Health Sciences
WHO: World Health Organization
DMFT: Decayed missing filled teeth
IRC: Institutional Review Committee
CI: Confidence interval
TMJ: Temporomandibular joint
CPI: Community periodontal index
LOA: Loss of attachment
